# A comprehensive study on changes in coastal hydrodynamics associated with cyclonic activity

**DOI:** 10.1038/s41598-024-58575-w

**Published:** 2024-05-22

**Authors:** Nada M. Salama, Kareem M. Tonbol, Ahmed ElKut, Mohamed ElBessa, Vassiliki Kotroni

**Affiliations:** 1 Department of Oceanography, Faculty of Science, Alexandria University, Alexandria, Egypt; 2 College of Maritime Transport and Technology, Arab Academy for Science, Technology and Maritime Transport, Abu-Qir, Alexandria Egypt; 3 Department of Hydrodynamics, Coastal Research Institute, National Water Research Center, Alexandria, Egypt; 4 Institute of Environmental Research and Sustainable Development, National Observatory of Athens (NOA), Athens, Greece

**Keywords:** Mediterranean cyclone, Extreme event, Energy transport, SWAN model, Climate sciences, Atmospheric science, Atmospheric dynamics

## Abstract

A Mediterranean cyclone is a weather phenomenon capable of producing extremely severe conditions, including heavy rainfall and strong winds. Between March 24 and 26, 2023, a cyclone passed along the western Egyptian Mediterranean coast, spanning three days. This paper aims to investigate the cyclone's impact on wave characteristics, focusing particularly on simulating changes in the energy transported from wind to waves during its passage, which constitutes the core objective of this study. The research methodology involved collecting meteorological and hydrodynamic data over five days from March 23 to 27, 2023, utilizing databases of the Bologna Limited Area Model (BOLAM) and the General Bathymetric Chart of the Oceans (GEBCO). This data, combined with field data for model calibration and validation, was analyzed using the Simulating the WAves Nearshore (SWAN) model packaged within the Delft 3D hydrodynamical model, integrated with other data manipulation tools. (SWAN) demonstrated the ability to simulate energy transport during extreme weather events along the coastal area with high resolution, up to 500 m. The results indicate a significant increase in significant wave height, reaching up to 2.5 m, and disturbances in wind direction, with velocities exceeding 10 m per second. These conditions pose risks to the infrastructure in some cities along the study area and have severe impacts on coastal communities. A notable finding from the simulations is the excess energy transport, which reached up to 12,000 watts per meter over the sea surface during the cyclone. Furthermore, calibration and validation results affirm the (SWAN) model's capability to accurately study wave characteristics.

## Introduction

The Mediterranean Sea, spanning 2.5 million square kilometers and bordering Europe, Africa, and Asia, exhibits diverse topography and numerous islands. It connects to the Atlantic, Black, and Red Seas, experiencing limited rainfall and high evaporation. Geographic challenges include the 4800-m-high Alps, influencing water and atmospheric circulation. Thornes^1^ and Lionello^2^ underscore this complexity, providing insights for academic research on the distinctive features of the Mediterranean, while more recently Michaelides et al.^3^ reviewed the processes that are significant in the weather and hydro-meteorological hazards in the area. Given the Mediterranean's unique position, which introduces the potential for subtropical or tropical-like storms and its location downstream of the North Atlantic storm track that enhances and amplifies the power and severity of cyclones formed in this region, numerous efforts are underway to comprehend cyclone behavior and impacts^4^.

Mediterranean cyclones, emerging as severe events causing damage to human life, generally exhibit lower intensities, smaller dimensions, and briefer durations compared to mid-latitude cyclones forming over open oceans, yet they frequently exert significant influence, playing a crucial role in the majority of extreme weather events, including precipitation and wind patterns^4–7^.

Recent studies have highlighted the increasing impact of Medicanes on coastal areas, particularly in the Mediterranean region. Scicchitano^8^ found that Medicanes can cause more significant flooding than seasonal storms, with Medicane Zorbas in 2018 reaching a surge of 0.8–1.2m above mean sea level. Nastos^9^ and Bakkensen^10^ emphasized the high activity of Medicanes during autumn and winter, with Italy suffering the highest expected damages at $33 million annually.

A cyclone, a low-pressure system primarily originating from baroclinic instability, sees the genesis of intense cyclones in the Mediterranean initiated by deviations in the polar jet stream. This leads to the infiltration of air masses with high potential vorticity (PV) into the region. Such intrusion induces baroclinic instability similar to extratropical cyclones observed in open oceans. Rossby wave breaking (RWB) frequently serves as a precursor, often resulting in the formation of deep cyclones. The cyclogenesis environment is marked by significant horizontal shear, guiding Mediterranean cyclones through a standardized baroclinic life cycle. The intrusion of the polar jet into the Mediterranean may coincide with the subtropical jet, mutually accelerating both jets. Mediterranean cyclone formation occurs along lower-tropospheric temperature gradients influenced by disparities in land-sea temperatures.

The organization of air masses within the cyclone, particularly the warm and cold conveyor belts and dry air intrusion, plays a pivotal role in shaping its life cycle and determining its variable impact^6,11–14^.

There are various types of Mediterranean cyclones, including Medicanes, which are infrequent mesoscale cyclones. They were initially identified based on their visual resemblance to tropical cyclones in satellite imagery, characterized by a central "eye" and spiral cloud bands encircling the core, along with robust winds in the eyewall. This similarity extends to features commonly observed in both tropical cyclones and polar lows^4,15–20^. Regarding Sea Surface Temperature (SST), Medicanes emerge over sea surface temperatures ranging from 15 to 23 °C, which is below the established threshold for tropical cyclones (26.5 °C). The intrusion of cold air into the upper troposphere facilitates an efficient conversion of thermal energy into kinetic energy, even when SST is relatively low, as reported by Tous et al.^20^. North African Cyclones, the second type, primarily originate on the sheltered side of the Atlas Mountains, displaying a distinct climatological peak in the spring season and forming over the arid territories of North Africa^21^. Vb cyclones, as delineated by van Bebber in 1891^22^, are characterized as low-pressure systems that are most prevalent during the spring season. According to Messmer et al.^23^ these cyclones propagate in a northeastward direction from the western Mediterranean Sea to Central Europe, traversing northern Italy and moving to the left of the Alpine ridge. Mediterranean explosive cyclones, mentioned by northwest Kouroutzoglou et al.^24^ and Reale et al.,^25^ and marked by swift intensification, occur approximately 5–6 times each year, reaching their zenith between November and March. They primarily originate within the region but occasionally intrude from the northwest. "Daughter cyclones," or secondary cyclones, emerge on the outskirts of pre-existing cyclones. Ziv et al.^26^ discovered that the majority of Mediterranean daughter cyclones originate from parents within the same region, exhibiting unique features.

Furthermore, there is a greater abundance of research concentrating on the western Mediterranean in comparison to studies centered on the eastern Mediterranean or the coastlines of Africa as reported by^4^. However, it is well known that in a 2015 study, Almazroui et al.^27^ identified 1992 cyclone tracks over the East Mediterranean region using six-hourly sea level pressure fields taken from the National Center for Environmental Prediction and the National Center for Atmospheric Research (NCEP/NCAR) reanalysis spanning from 1958 to 2010. A classification method was developed to classify the long-track cyclones into four key routes. Additionally, two other routes were considered, which were classified as either stationary or having a short track length. This study revealed that the East Mediterranean region has three main cyclogenesis areas and four main cyclosis areas.

Furthermore, numerous questions persist regarding the behavior of cyclones and various interactions, all of which remain subjects of ongoing research. Additionally, the passage of a cyclone over the sea leads to changes in the hydrodynamics of the seawater, all these factors increase the importance of this research. The area under investigation is western Egyptian Mediterranean coast. The fundamental principles of coastal hydrodynamics, as documented by Iskander et al.^28^ and Salama et al.^29^, underscore waves as a primary driver of beach dynamics, predominantly initiated by offshore winds. Additionally, waves significantly influence the nearshore current system. Upon scrutinizing the available wave data spanning from 2015 to 2018, it is evident that the significant wave height (Hs) within the study area consistently falls within the range of 0.5 m to 1.4 m for the majority of the year. Noteworthy maximum wave heights are recorded offshore and nearshore at 5.1 m and 4.4 m, respectively. Furthermore, the significant wave height during winter months surpasses that observed in the summer. The temporal analysis of wave periods reveals an annual fluctuation between 4 and 6 s, with a peak maximum wave period reaching approximately 10 s. The prevailing wave direction, predominantly oriented to the northwest, exhibits a perpendicular approach to the shoreline. Concurrently, the predominant wind influence in 2018 emanates from the west-northwest, with wind speeds fluctuating between 4 and 8 m/s. These insights contribute to a nuanced comprehension of the intricate interdependencies among significant wave height, wave period, wave direction, and wind direction in the coastal hydrodynamic context during the specified study period Salama et al.^29^.

Various studies have elucidated the generation and enhancement of wind-driven waves over decades, encompassing practical experiments and theoretical hypotheses. Early contributions include the initiatives by Jeffreys^30^ and^31^, Phillips^32^, Miles^33^, and later studies by Hristov et al.^34^, and Cavaleri et al.^35^. As reported by Deigaard and Nielsen^36^ insufficient focus has been directed towards understanding the intricacies of water motion within developing waves. This pertains to the mechanisms responsible for the transfer of energy and momentum to the waves, their distribution along the vertical axis, and other research points. Additionally, there are numerous research questions related to air-sea interactions, particularly during extreme conditions.

**Figure 1:**
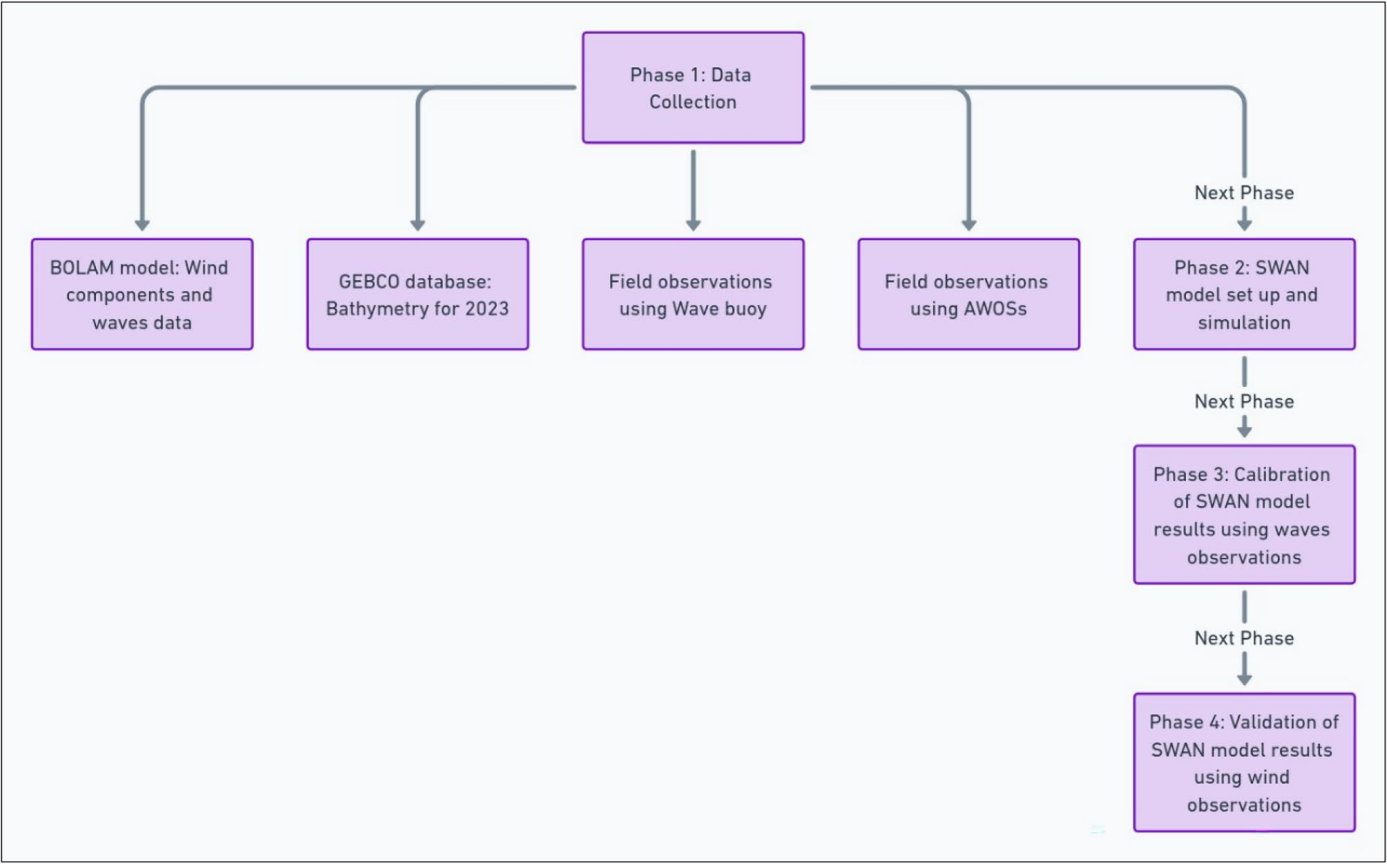
Framework for the research methodology and design.

**Figure 2:**
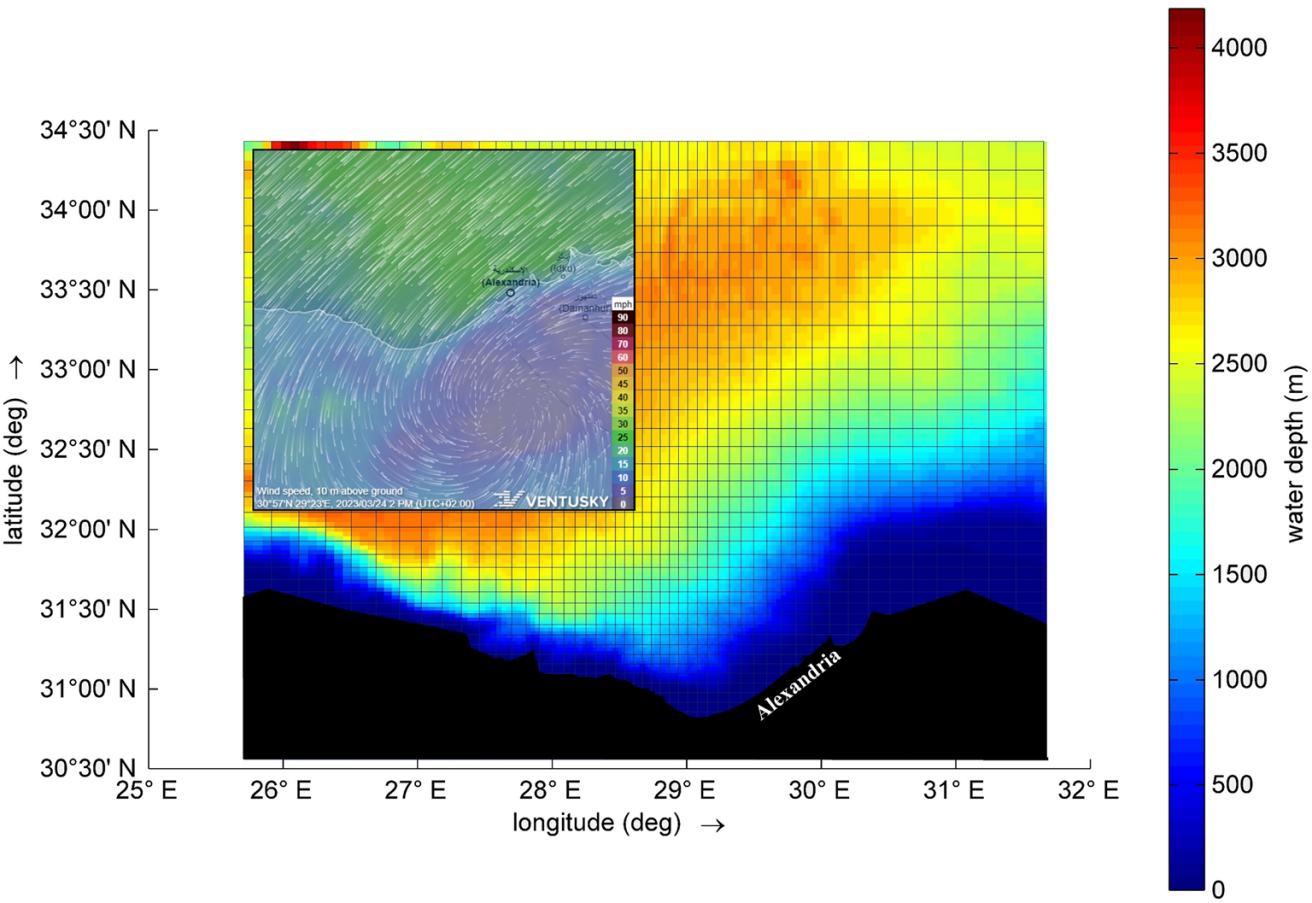
The water depth of the study area is implemented using the Delft 3D-hydrodynamical model, which constitutes the fundamental step in model setup for this work.) Upper left image derived from the Ventusky website shows a screenshot for the cyclone, which is based on ECMWF data (https://www.ventusky.com/?p=30.93;31.59;7&l=wind-10m&t=20230324/12&m=icon&w=strong).

Generally, the hydrodynamics of the Mediterranean surface water, including waves, water currents, sediment transport, and coastal processes, are influenced by the passage of cyclones. This impact arises from changes in sea surface temperature, atmospheric pressure, wind forces, shear stress, and more. On March 24, 2023, a cyclone formed over the western Egyptian Mediterranean coast, persisting for three days. It affected weather conditions, wave heights, and resulted in temporary coast inundation. This study aims to investigate changes in wave characteristics and energy transport as cyclones move across the study area. The research design is depicted in Fig. 1. Furthermore, Fig. 2 illustrates the area under investigation and water bathymetry, with Alexandria being the most affected city during such events. In this study, the introduction is presented in “Introduction” section, while “Materials and research design” section encompasses the materials and research design. “Methods” section outlines the methodology employed. Results and discussion are combined in “Results and discussion” section, with conclusions and recommendations provided in “Conclusions and recommendations” section.

## Materials and research design

The full design of this work, beginning with phase (1) of data collection, which showcases a variety of data used in the initial and boundary conditions. Additionally, phase (2) encompasses setting up and running the SWAN model on high resolution domain, analyzing results, comparing observations with model results through model calibration, and concludes with model validation in phases 3 and 4, as depicted in the research methodology framework, Fig. 1.

### Modeled data

The water depth is obtained using a contour map produced by general bathymetric charts for the ocean (GEBCO), then used in setting up the hydrodynamical model, Fig. 2. The wind data were provided from the operational weather forecasting datasets of the METEO Unit at the National Observatory of Athens (NOA) and included 5 days from 23 up to 27 March 2023 with a temporal resolution of 1 h and a spatial resolution of 0.06 × 0.06°. The numerical weather prediction model used is BOLAM which is run for operational weather forecasting at NOA since 2000. Important elements for BOLAM as reported by Lagouvardos et al.^37,38^ consist of hydrostatic primitive equations, parameters including surface pressure, wind elements, potential temperature, specific humidity, and microphysical parameters. It utilizes an Arakawa-C grid in vertical σ coordinates. The approach involves a forward–backward 3D Eulerian advection method, semi-Lagrangian advection for hydrometeors, and a split-explicit time scheme for gravity modes. The model includes physical representations such as dry adiabatic adjustment, radiation interaction with clouds, vertical diffusion based on Richardson number, surface thermal and water equilibrium, an explicit microphysical mechanism, and a convective parameterization strategy.

**Figure 3:**
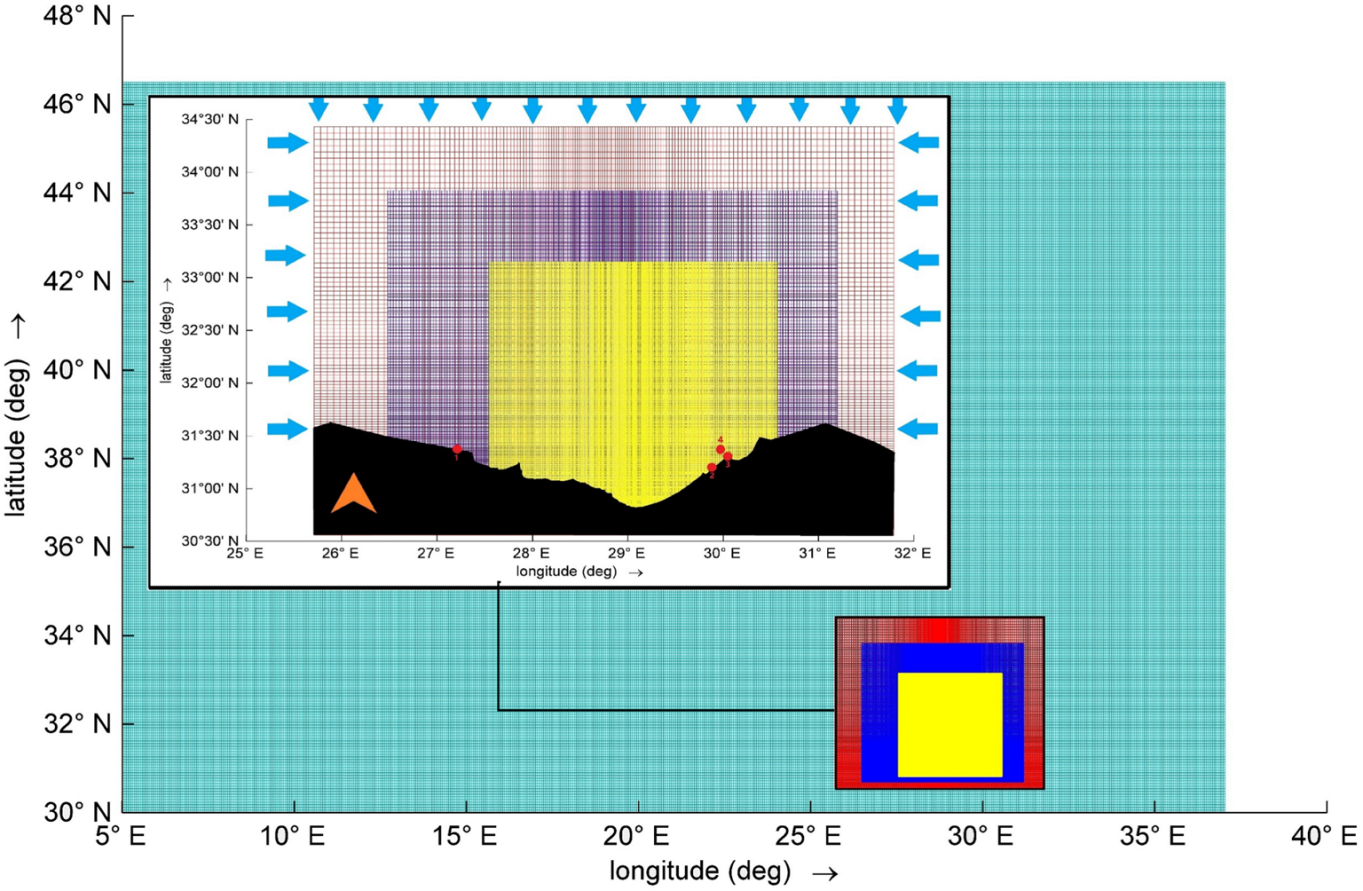
The wind grid (only the green layer and the embedded nested grids for the SWAN model), where the coarser SWAN grid is in red, the middle-resolution SWAN grid is in blue, and the finest grid is in yellow. Additionally, the blue arrows indicate the open boundaries, and the red dots mark the positions of field data.

The wind datasets, consisting of u and v components, cover the Mediterranean area as shown in Fig. 3. They have a resolution of 0.06 × 0.06° and are measured at an elevation of 10 m above mean sea level. This coverage spans between 30° N and 48° N, and 5° E and 40° E. Additionally, a wave dataset was obtained for the study area, indicated in Fig. 3 by the coarser red-colored domain. This wave dataset shares the same resolution of 0.06 × 0.06 degrees and extends between 31° N and 34° 30' N, and 26° E and 32° E.

#### Waves observations by SD4W wave gauge

The S4DW wave gauge, located at a depth of 14 m in Abu Quir, utilizes pressure measurements at the sea surface to assess wave characteristics. Situated in relatively shallow waters and anchored to the sea floor, this gauge's reliance on surface pressure measurement may result in inaccuracies, particularly in detecting high-frequency waves. It is worth noting that the same S4DW gauge is mentioned in the work of Elkut et al.^39^. Additionally, wave parameters such as wave height (m), wave period (s), and wave direction were recorded during February 10–12, 2023. The geographic location is depicted in Table 1 and Fig. 3 by red dots.

**Table 1:** Elevations and positions of Coastal Meteorological AWOSs and the wave gauge SD4W.

IN	Station name	Height in (m) related to MSL	Latitude (°N)	Longitude (°E)
1	Marsa Matruh (AWOS)	20 above	31° 21ʹ 34″	27° 14ʹ 43″
2	Ras El Tin (AWOS)	22 above	31° 11ʹ 50″	29° 51ʹ 49″
3	Abu Qir (AWOS)	26 above	31° 19ʹ 55″	30° 5ʹ 6″
4	Abu Quir (SD4W)	14 below	31°21ʹ 3.71″	30° 6ʹ 20.46″

The identification numbers (IN) 1 to 4 are also included in relation to Fig. 3. Additionally, the temporal resolution for all devices is 1 h for the total duration of 3 days, with 72 records per station.

#### Wind observations

Automated Weather Observing Systems (AWOSs) were employed to meet the study's requirements at three locations: Marsa Matruh, Ras El Tin, and Abu Qir, as highlighted in Table 1 and Fig. 3 by red dots. The recorded measurements include near-surface temperature (T2m), wind speed (W10) and direction, and sea-level pressure (P). These AWOSs were installed and maintained in accordance with WMO regulations. Meteorological parameters such as wind speed (in knots) and direction (in degrees), air temperature (in °C), and sea-level pressure (in hPa) were calibrated to WMO standard heights. To ensure data integrity, each AWOS is equipped with two systems. Wind speed and direction are measured ultrasonically by the WXT520 with Vaisala’s advanced WINDCAP sensor. Additionally, the barometric pressure and temperature sensors are housed in a ventilated chamber. It is worth mentioning that Automated Weather Observing Systems (AWOSs) were discussed in the work of El-Geziry et al.^40^.

## Methods

### SWAN model setup configuration

Regarding the computational domains for the scenarios applied during this work, initially, the SWAN model was developed on nested grids, as shown in Fig. 3. There are three nested grids used to simulate the waves down to 500 m, representing a high-resolution grid or fine grid. Followed by generating the bathymetry map associated with each grid based on GEBCO data. Considering the resolution of the coarse domain varies between 2300 and 6500 m including 18,150 nodes, the resolution of the intermediate domain between 1300 and 2300 m including 34,126 nodes, and the resolution of the fine domain between 500 and 750 m including 115,255 nodes.

In running the SWAN model, boundary conditions involve using a coarse grid for input data, Fig. 3. The boundaries of the middle and fine grids receive values from the coarser domain. The open boundaries consist of the north, east, and west boundaries, with the south boundary defined by land. Zero mean sea level is applied to all active boundaries. Regarding wind, there are four open boundaries due to winds blowing from all directions. Each point derived from the BOLAM model is positioned to its original location within the SWAN domain.

SWAN (**S**imulating the WAves Nearshore) version 40.73CDE, crafted by Delft University of Technology, is employed in this study. SWAN model is a third-generation wave model which takes into account the meteorological factors including wind growth during cyclones. SWAN can solve the transmission formula with no constraints on the spectrum structure of the wave energy, and computes the development of wind waves in coastal locations with shallow water. Wind input and nonlinear wave exchange are examples of these phenomena. Six processes have an effect on source and sink terms and are all modelled.

The term $\left(S(\sigma ,{\theta }_{\omega };x,y,z)\right)$ depicts the source and sink components.

1 $$S\left(\omega , {\theta }_{\omega }\right)={S}_{in}\left(\omega , {\theta }_{\omega }\right)+ {S}_{n{l}4}\left(\omega , {\theta }_{\omega }\right)+ {S}_{n{l}3}\left(\omega , {\theta }_{\omega }\right)+{S}_{\omega cap}\left(\omega , {\theta }_{\omega }\right)+{S}_{bot}\left(\omega , {\theta }_{\omega }\right)+{S}_{br}\left(\omega , {\theta }_{\omega }\right)$$

Where ${S}_{in}$ denotes the wave development as a consequence of the wind input, The nonlinear transportation of wave energy through three-wave and four-wave exchange is expressed by the second and third constituents, respectively. The last three terms, white-capping, bottom friction, and depth induced wave breaking, indicate wave decay and dissipation, respectively. SWAN is a renowned spectral wave model capable of solving the transport equation without constraints on the form of the wave energy spectrum.

### Simulation scenarios

Applying the SWAN model to the study area involved conducting two scenarios to illustrate variations in wave characteristics, dissipation, and energy transport during a cyclone. The first scenario simulates the impact of a cyclone on the water hydrodynamics in the study area, while the second scenario replicates the normal hydrodynamics. Considering that the conditions were identical for both scenarios, except for excluding wind growth in the second scenario.

### Wind forcing of wave generation and energy transfer

Nielsen et al.,^41^ stated that airflow interacts with water waves through surface stresses, inducing pressure $${p}^{+}$$ and shear stress $$\overset{\lower0.5em\hbox{$\smash{\scriptscriptstyle\smile}$}}{\tau }$$. Wind transfers energy to waves if stresses align with the wave's wavelength for effective movement. Wind forcing of wave growth is governed by Eq. (2). To enable the pressure applied to the water surface to perform work, the surface needs to undergo movement perpendicular to its own direction, the work rate done by pressure $${p}^{+}$$ per unit area equals the pressure multiplied by the change in volume divided by the unit surface area $${p}^{+}\frac{\partial \eta }{\partial t}$$ also, the progressive wave of surface elevation given by $$\eta$$, the vertical motion is shifted by phase angle equals $$\frac{\pi }{2}$$ with respect to the surface elevation, $$\omega$$ is the cyclic frequency and k is the wave number.

2 $$\eta ={\text{acos}}(kx-\omega t)$$

Deigaard and Nielsen^36^ reported that, to maintain the energy balance and enhance wave energy per unit area, the energy (E) must be devoid of or have a shortage in energy dissipation. It should equal the rate of work exerted by surface pressure (Eq. 3), where the use of an overbar denotes averaging over a brief time interval associated with a wave's period.

3 $$\frac{dE}{{d}_{t }}= \stackrel{-}{-p+\frac{\partial \eta }{\partial t}}$$

### Model calibration

Williams and Esteves^42^ reported that calibration is achieved through a comparison between measured field data (observations) and model outcomes (modeled data). This study primarily focuses on changes in wave characteristics. Therefore, the model's wave outcomes (M) will be calibrated using wave observations (O) collected by a wave gauge (SD4W). Error statistic will be achieved by calculations of the Correlation coefficient expressed as (R), the Root Mean Square Error (RMSE), BIAS, Mean Absolute Error (MAE), and Scatter Index (SI). Due to complications in obtaining wave observations during the exact period of the cyclone which passed on March 24–26, 2023, the available dataset for measured waves was during February 10–12, 2023, where March and February are winter months. Additionally, the measured wave parameters that will be used for this part of the study are the significant wave height in meters and the corresponding wave period in seconds. Also, the following equations will be applied and scatter plots between each modeled and observed parameter.

4 $$R= \frac{\sum_{i=1}^{n}(\left({M}_{i}-\overline{M }\right)\left({O}_{i}-\overline{O }\right))}{\sqrt{\sum_{i=1}^{n}{\left({M}_{i}-\overline{M }\right)}^{2 }(\sum_{i=1}^{n}{\left({O}_{i}-\overline{O }\right)}^{2})}}$$

5 $$RMSE=\sqrt{\frac{1}{n } \sum_{i=1}^{n}{({M}_{i}-{O}_{i})}^{2}}$$

6 $$BIAS= \sum_{i=1}^{n}\frac{1}{n}({M}_{i}-{O}_{i})$$

7 $$MAE=\frac{1}{n}{\sum }_{i=1}^{n}\left|{M}_{i}-{O}_{i}\right|$$

8 $$SI= \frac{RMSE}{\frac{1}{n} \sum_{i=1}^{n}{M}_{i}}$$

### Model validation

As outlined in “Materials and research design” section, wind data were retrieved from the BOLAM numerical weather prediction model database in the form of components (u & v). These components were then utilized as inputs for the SWAN model to detect the effect of wind growth during the cyclone on the sea surface hydrodynamics, also enabling the computation of wind speed and velocity within the computational domains. In relation to this, the wind, treated as a variable, will be one of the SWAN model outputs, and it can be utilized for the validation process. Additionally, wind data recorded at three locations—Marsa Matruh, Ras Eltin, and Abu Quir—using Automated Weather Observing Systems (AWOSs) will be used as observations. The geographic locations are shown in Fig. 3 and Table 1. The process of validating SWAN results will undergo by applying equations from 4 to 8 and using scatter plots between wind observations and modeled wind data extracted from the SWAN model, typically at the same geographic locations as the AWOSs.

## Results and discussion

### Simulation of cyclone effects (first scenario)

To investigate how cyclones affect the hydrodynamics of surface water, we drill down into detail a specific cyclone event that impacted the western Egyptian Mediterranean coast in March 2023. This case study was meticulously chosen for its potential to unveil crucial insights into the vital interplay between cyclones and coastal hydrodynamics knowledge directly applicable to both predicting and mitigating coastal hazards. The March cyclone, a mature shallow system, bore a unique combination of characteristics intensity, trajectory, and alarming proximity to vulnerable communities presenting a rare opportunity to observe its effects on key aspects like wave characteristics and energy transfer. By meticulously analyzing this case study, specifically focusing on two-time stamps within the cyclone's peak intensity [2023-03-25 17:00:00] and [2023-03-25 23:00:00], we can inform the development of more accurate forecast models and refine coastal management strategies to bolster the resilience of vulnerable communities not only in this region but potentially beyond.

**Figure 4:**
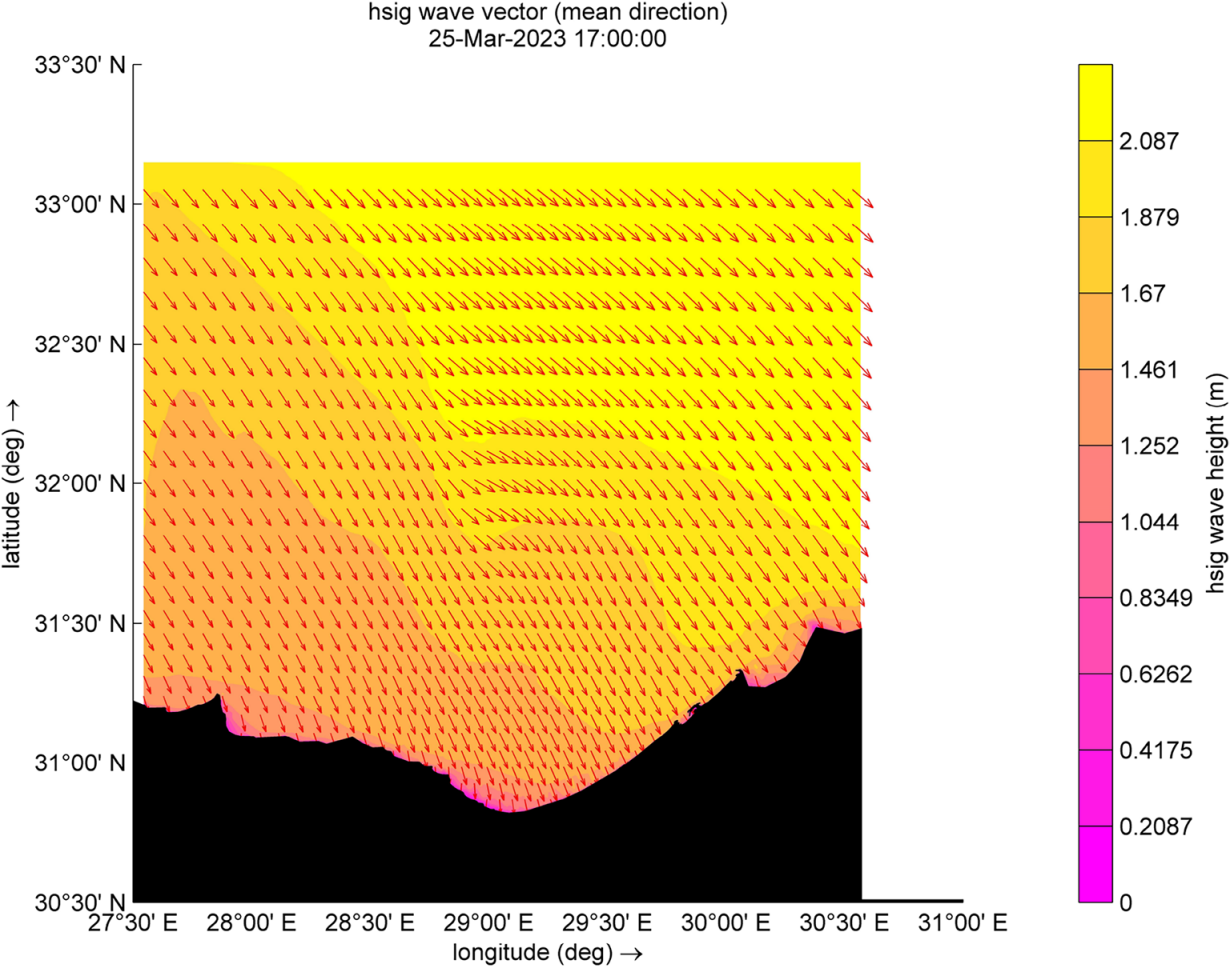
Distribution of significant wave height and wave direction (indicated by red arrows) during the cyclone event.

**Figure 5:**
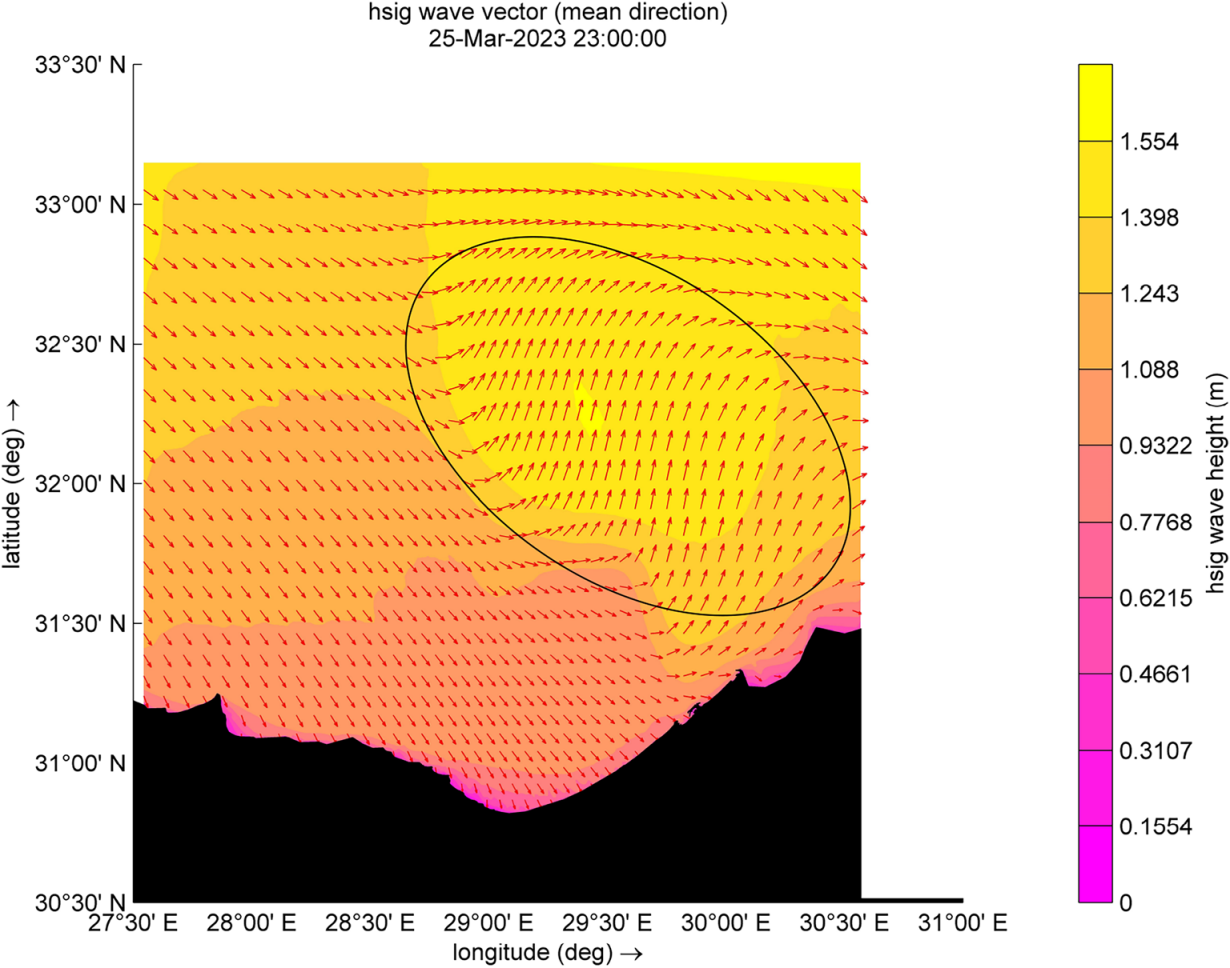
Distribution of significant wave height and wave direction (indicated by red arrows) during the cyclone event.

The results reveal a significant wave height of up to 1.2 m along the surf zone or breaker zone, as illustrated in Fig. 4. The waves approach the shoreline perpendicularly, considering that the coastline angle from the north is 47°. This alignment with northwest waves contributes to the development of a robust longshore current and the movement of sediment transport, can lead to erosion of the shoreline, which is agrees with studies of Iskander et al.^28^ and Salama et al.^29^. Consequently, some beaches may require backfilling after storms. Additionally, a group of waves reflects, resulting in a reduced significant wave height in both the surf zone and the offshore zone, as shown in Fig. 5. Moreover, the significant wave height (Hsig.) depicted in Fig. 4 is notably dangerous and abnormal, with the typical maximum Hsig. in the surf reaching up to 0.4 m during the normal conditions. Regarding the seasonal cycle of intense cyclones, various studies agree that cyclone formation is more frequent in winter and exhibits a relatively lower occurrence during the summer months, as indicated by Flaounas et al.^43^. Furthermore, for the study area, the winter season extends from November or December to March, emphasizing the susceptibility to cyclone formation during this period. Further, the wave direction is northwest, which is typical. However, at another time step, there are alterations and disruptions in the wave direction due to adverse weather conditions. Nevertheless, during this cyclone, there are unusual disturbances in the wind direction, opposing the wave direction in some areas, ultimately leading to a decrease in significant wave height (Hsig) in the surf zone. Conversely, there is an increase in Hsig. in the offshore area, coupled with the reflected waves resulting in the formation of a new fetch area, as shown in Fig. 5, with an oval shape.

**Figure 6:**
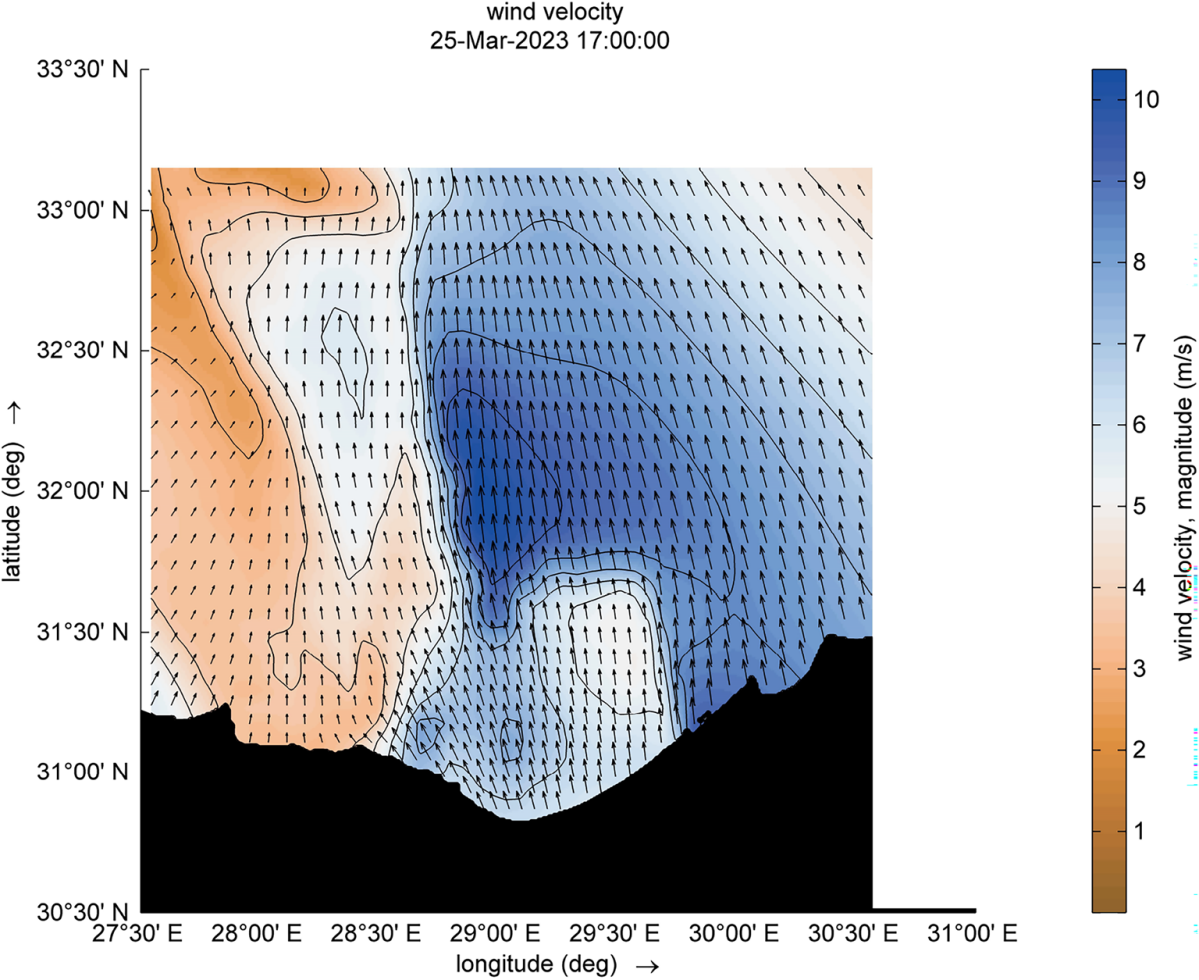
Distribution of wind magnitude and wind direction (indicated by black arrows) during the cyclone event.

**Figure 7:**
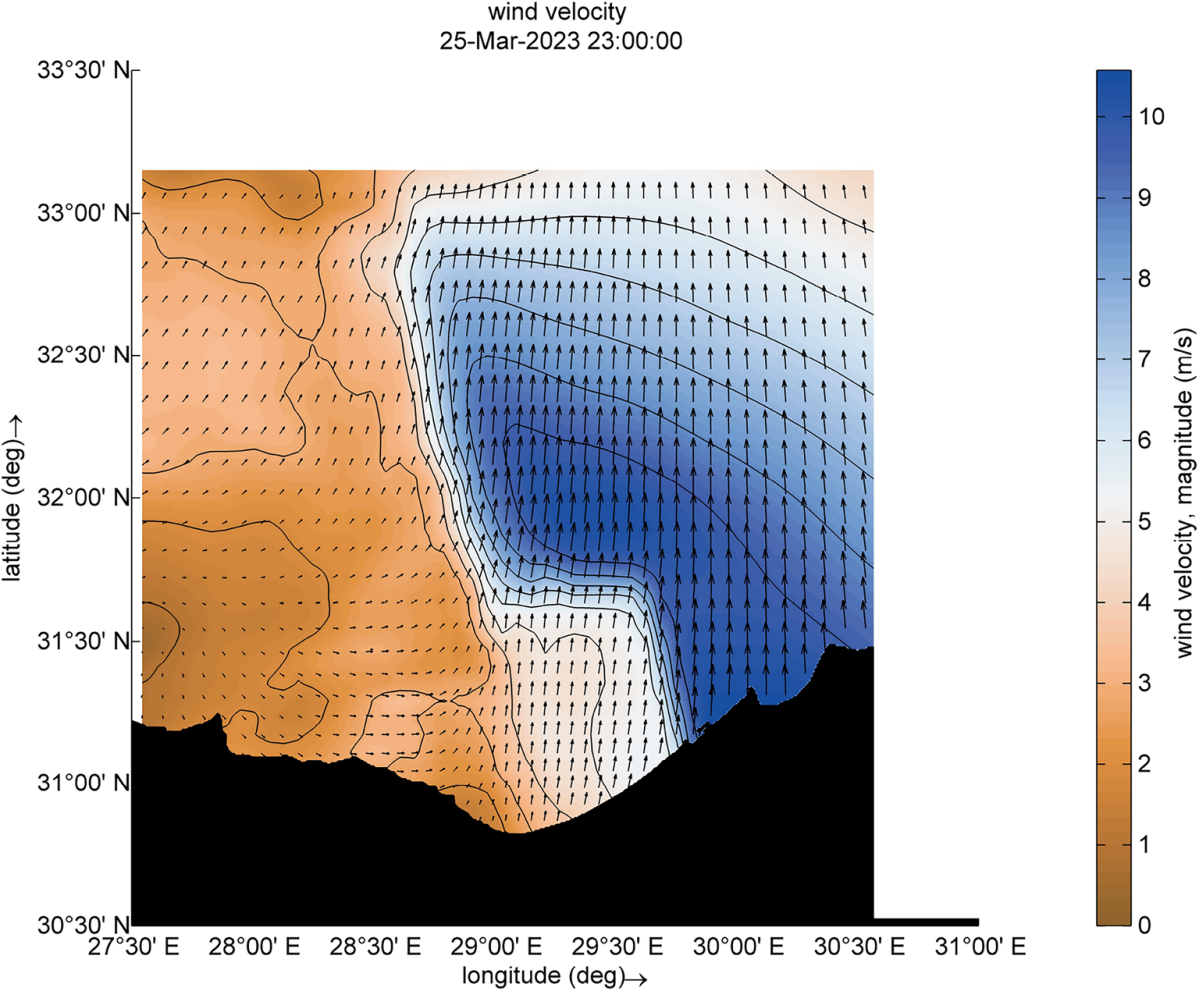
Distribution of wind magnitude and wind direction (indicated by black arrows) during the cyclone event.

For the two selected time steps ([2023-03-25 17:00:00] and [2023-03-25 23:00:00]), the wind velocity in the core of this cyclone is 10 m/s, as shown in Figs. 6 and 7. There is a slight change in direction between south and southeast. This change reflects the development of a growing wind field from almost the same direction over a specific period, resulting in the formation of the fetch area. A discernible contrast in wind velocities emerges between the eastern and western sectors of the cyclone. Specifically, the wind velocity registers at 10 m/s on the eastern side, juxtaposed with a velocity of 4 m/s on the western side. The outputs are agreed with studies of Elkut et al.^39^.

**Figure 8:**
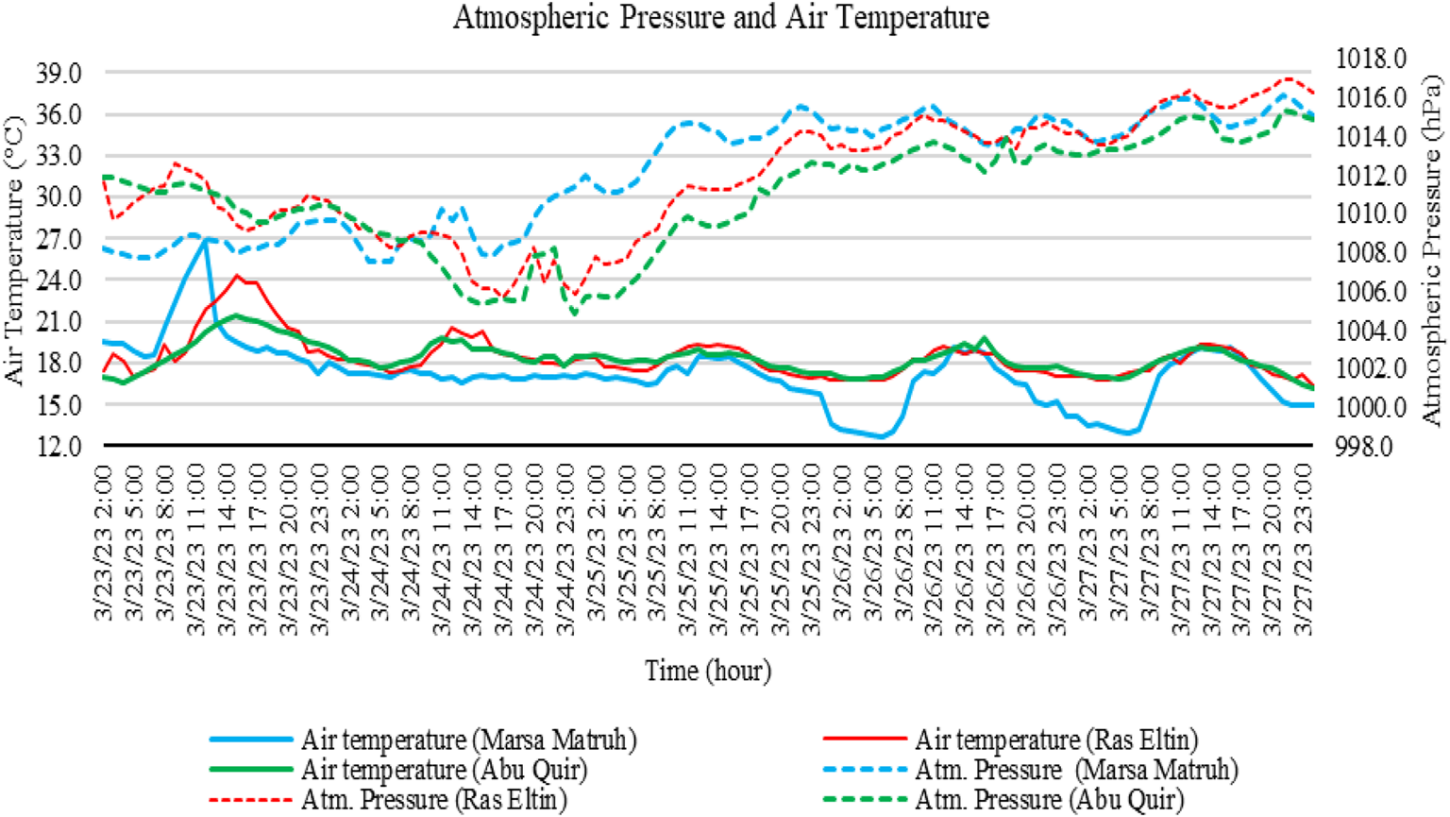
Time series for observations of air temperature (°C) and atmospheric pressure (hPa) at three stations—Marsa Matruh, Ras Eltin, and Abu Quir.

Additionally, complementary variables, integral to our analytical findings, encompass the results derived from the measured air temperature and atmospheric pressure data, as illustrated in Fig. 8. During the time step [2023-03-25 17:00:00], Marsa Matruh and Abu Quir represented the western and eastern sides, respectively. The air temperature and pressure readings were (17.6 °C, 1013.9 hPa) for Marsa Matruh and (18.4 °C, 1010.0 hPa) for Abu Quir. For the subsequent time step (2023-03-25 23:00:00), the readings were (15.9 °C, 1015.3 hPa) for Marsa Matruh and (17.2 °C, 1012.6 hPa) for Abu Quir. The results indicate resonance and a correlation between variations in air temperature, wind speed, and atmospheric pressure—characteristic conditions conducive to the formation of a small-scale Mediterranean cyclone, and these confirmed by studies of Tous et al.^20^. Furthermore, Fig. 2 is derived from an up-to-date prediction website based on the European Centre for Medium-Range Weather Forecasts database. The results show an anticlockwise circulation for the cyclone formed over Alexandria and typical wind speeds.

**Figure 9:**
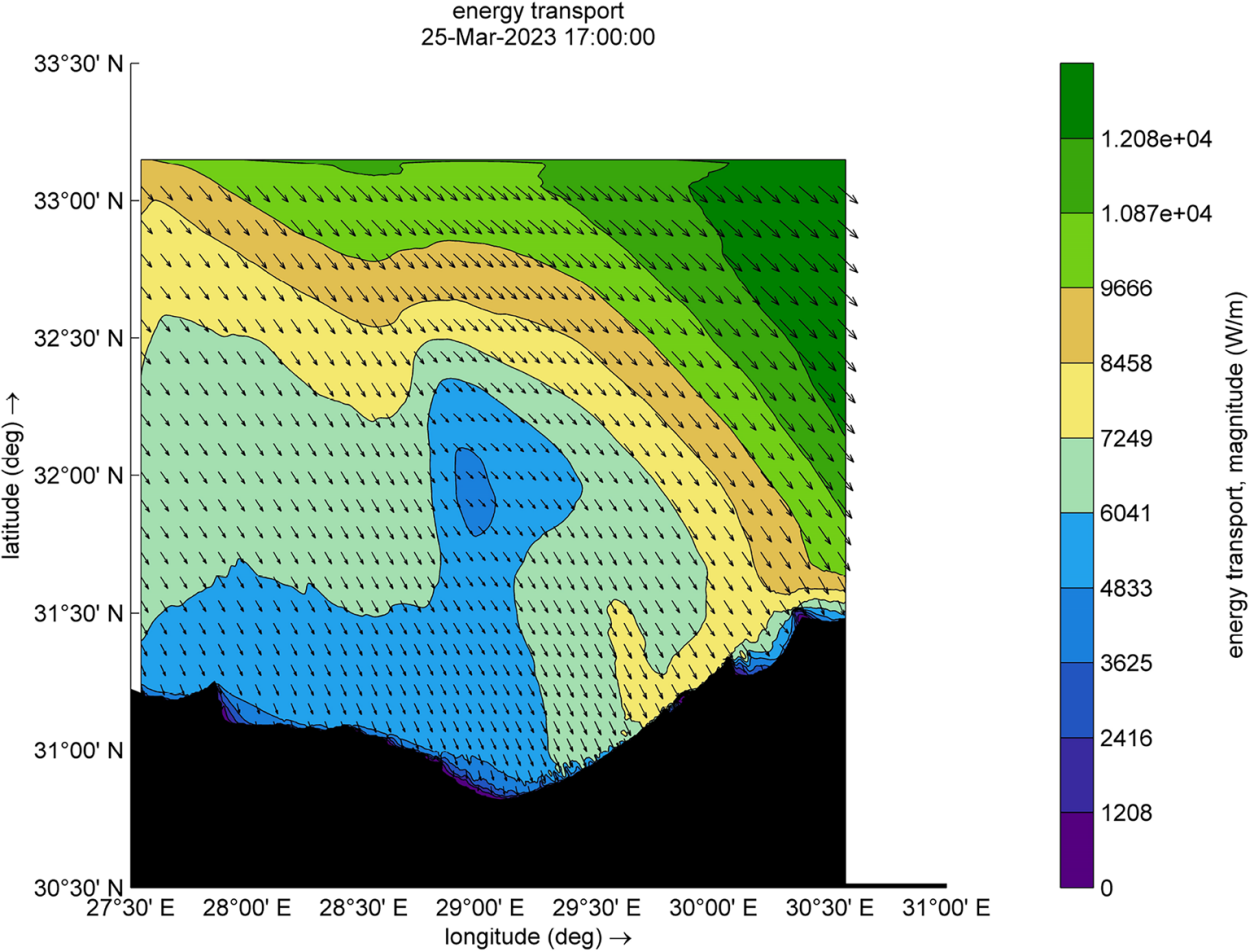
Distribution of energy magnitude and direction (indicated by black arrows) during the cyclone event.

**Figure 10:**
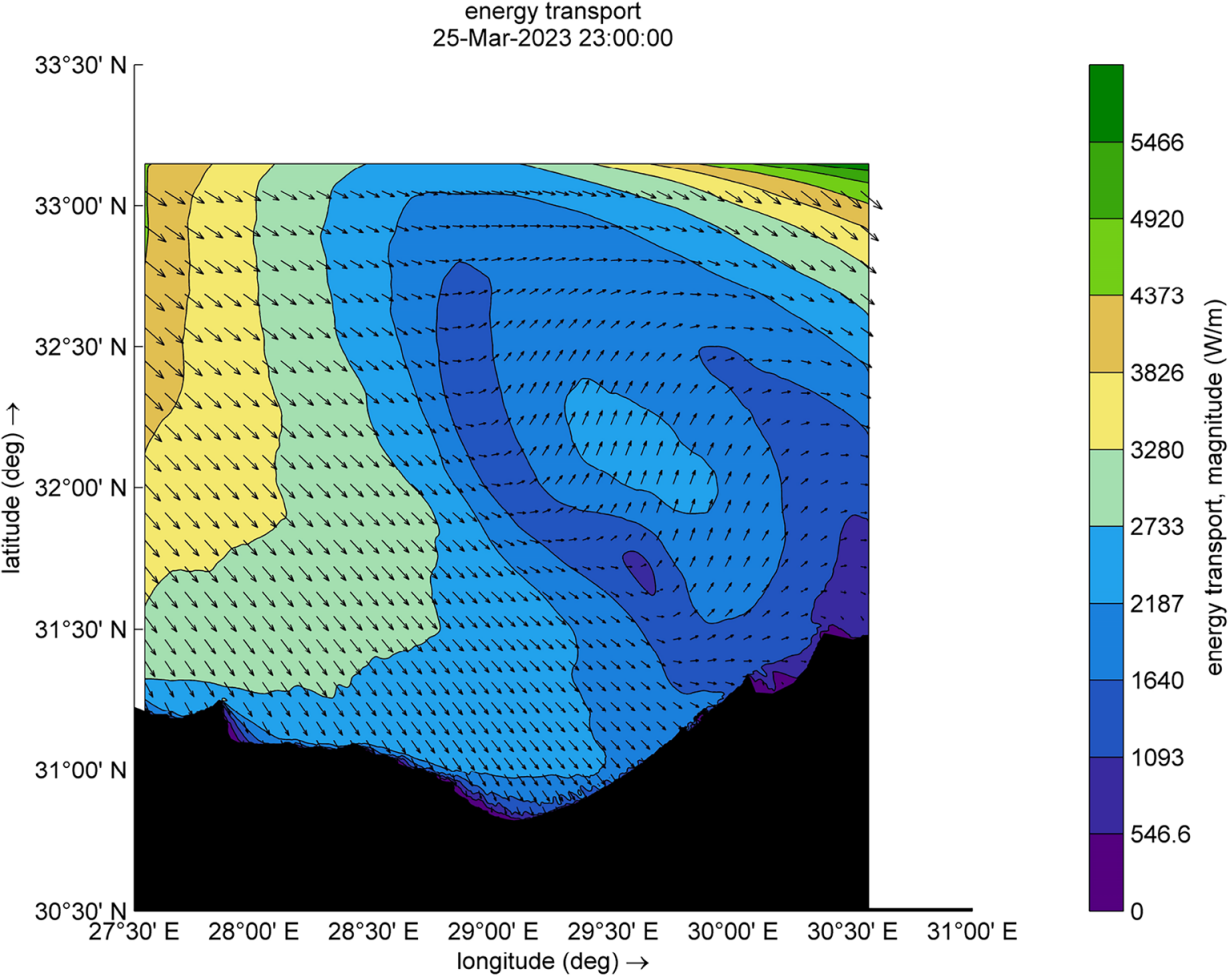
Distribution of energy magnitude and direction (indicated by black arrows) during the cyclone event.

**Figure 11:**
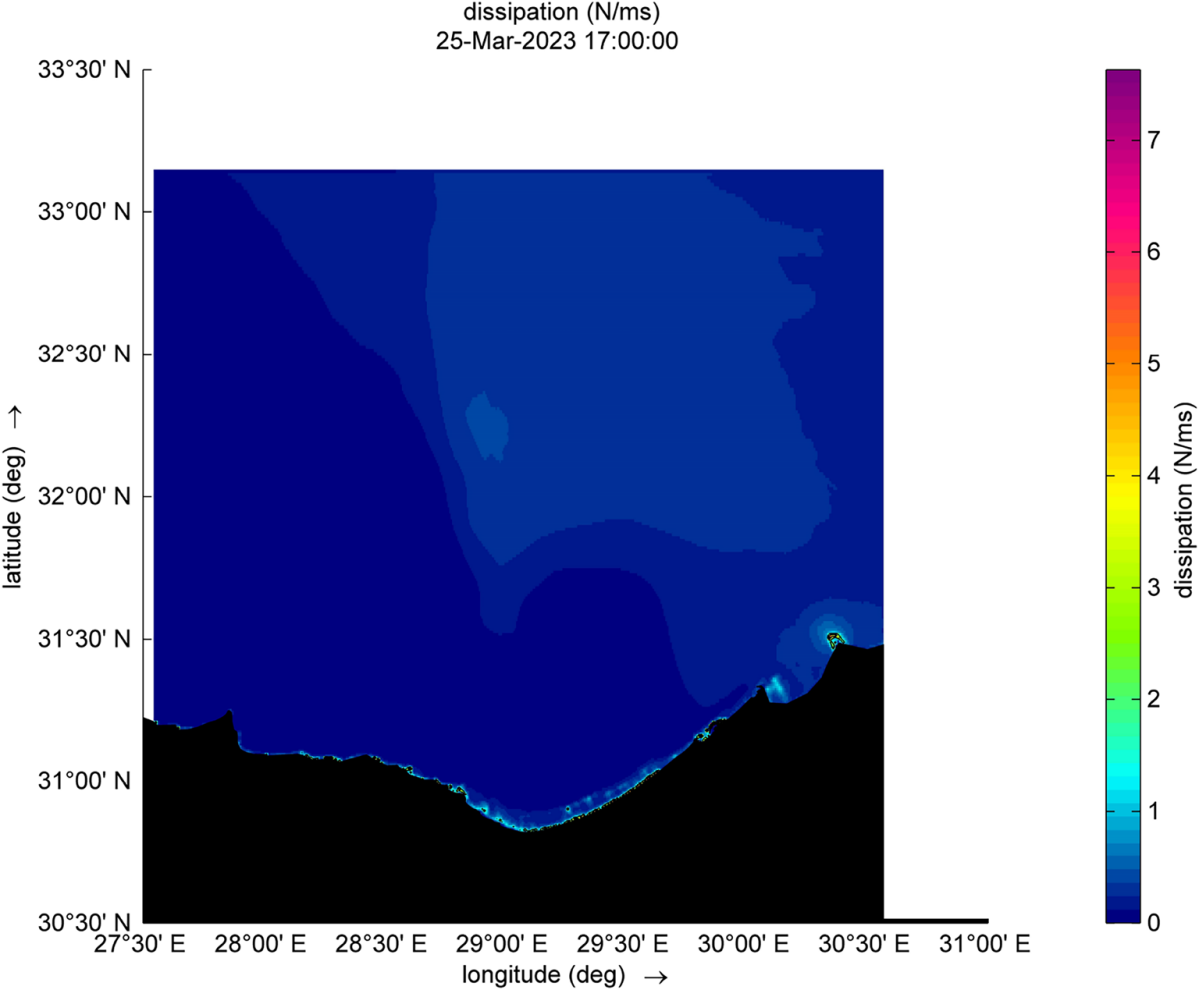
Dissipation during the cyclone event.

**Figure 12:**
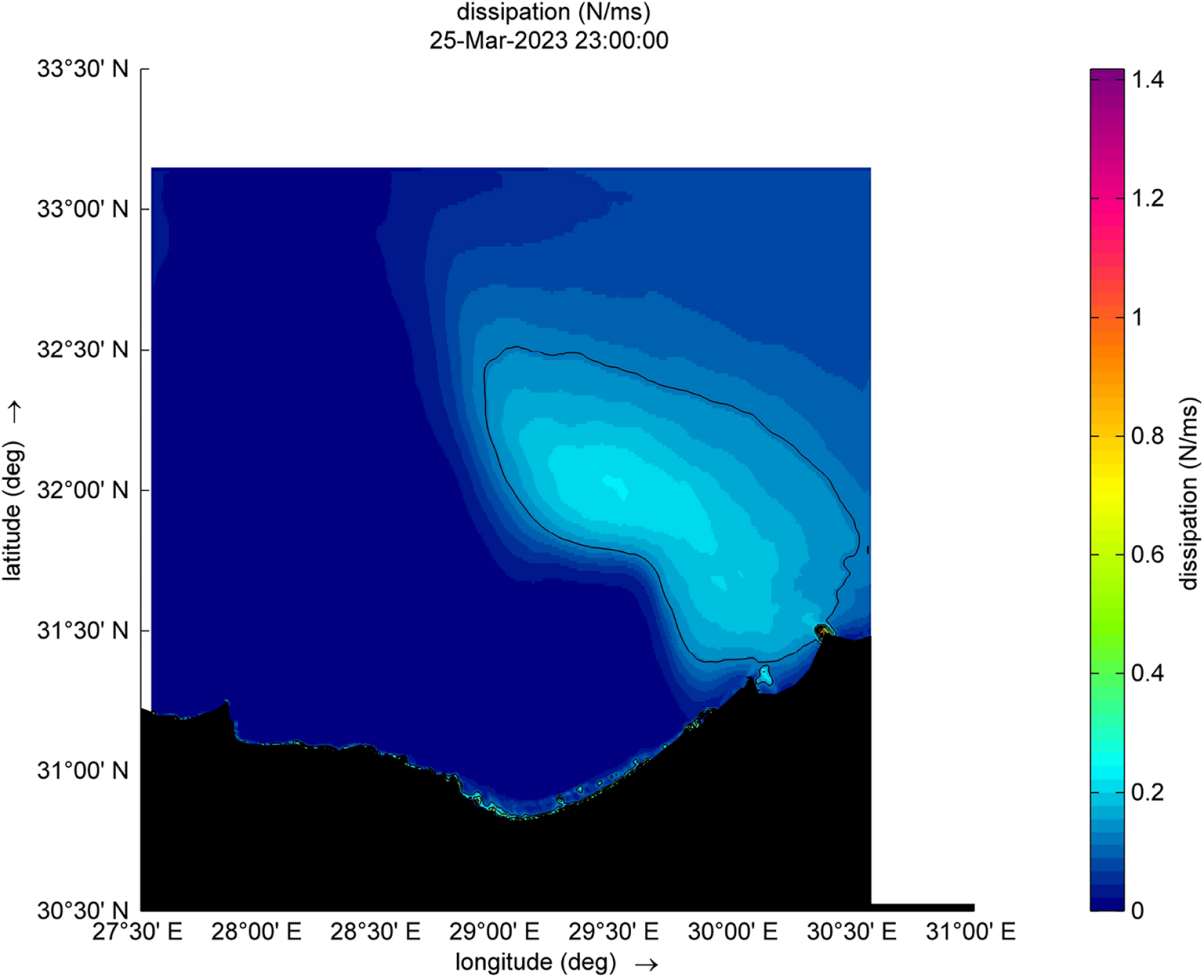
Dissipation during the cyclone event.

**Figure 13:**
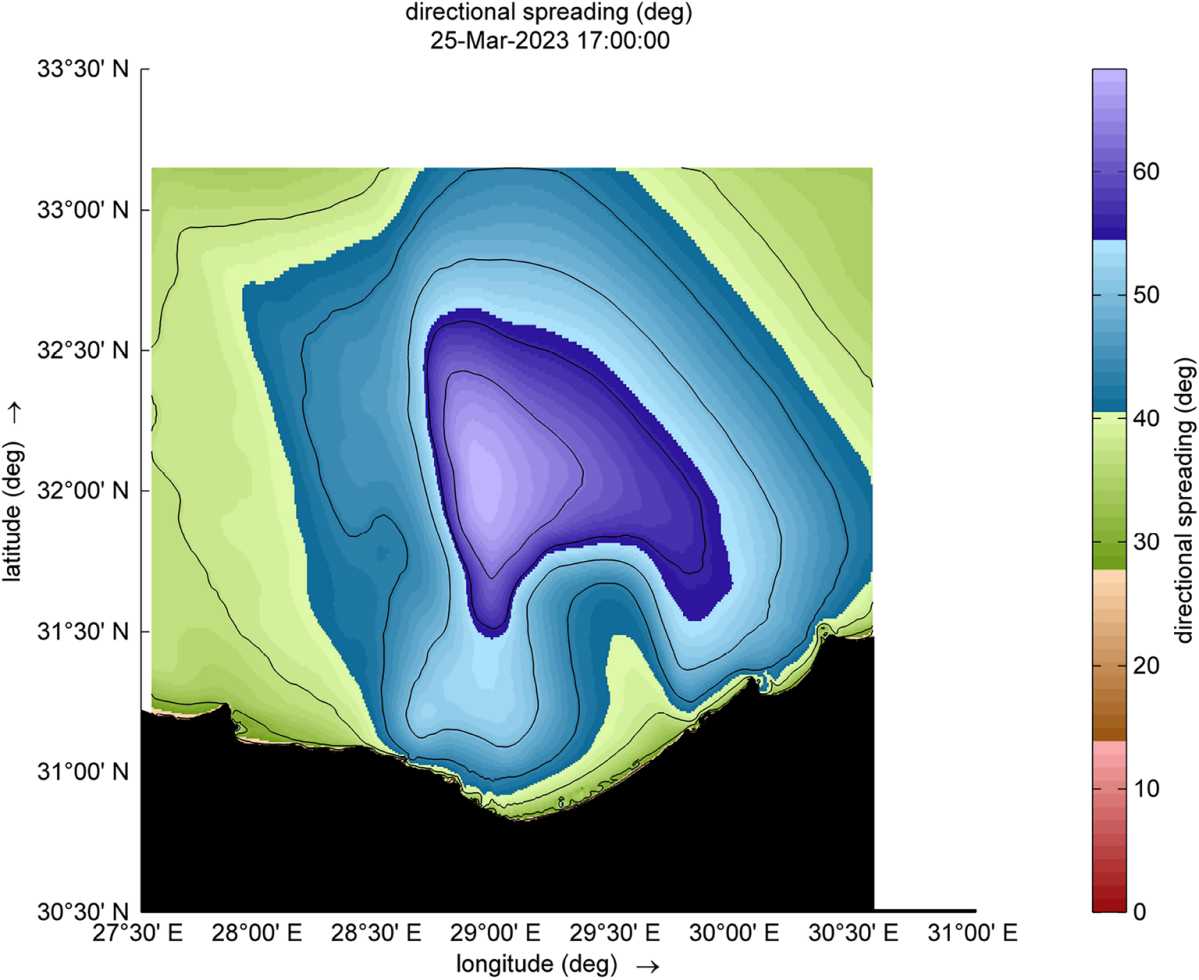
Directional spreading during the cyclone event.

**Figure 14:**
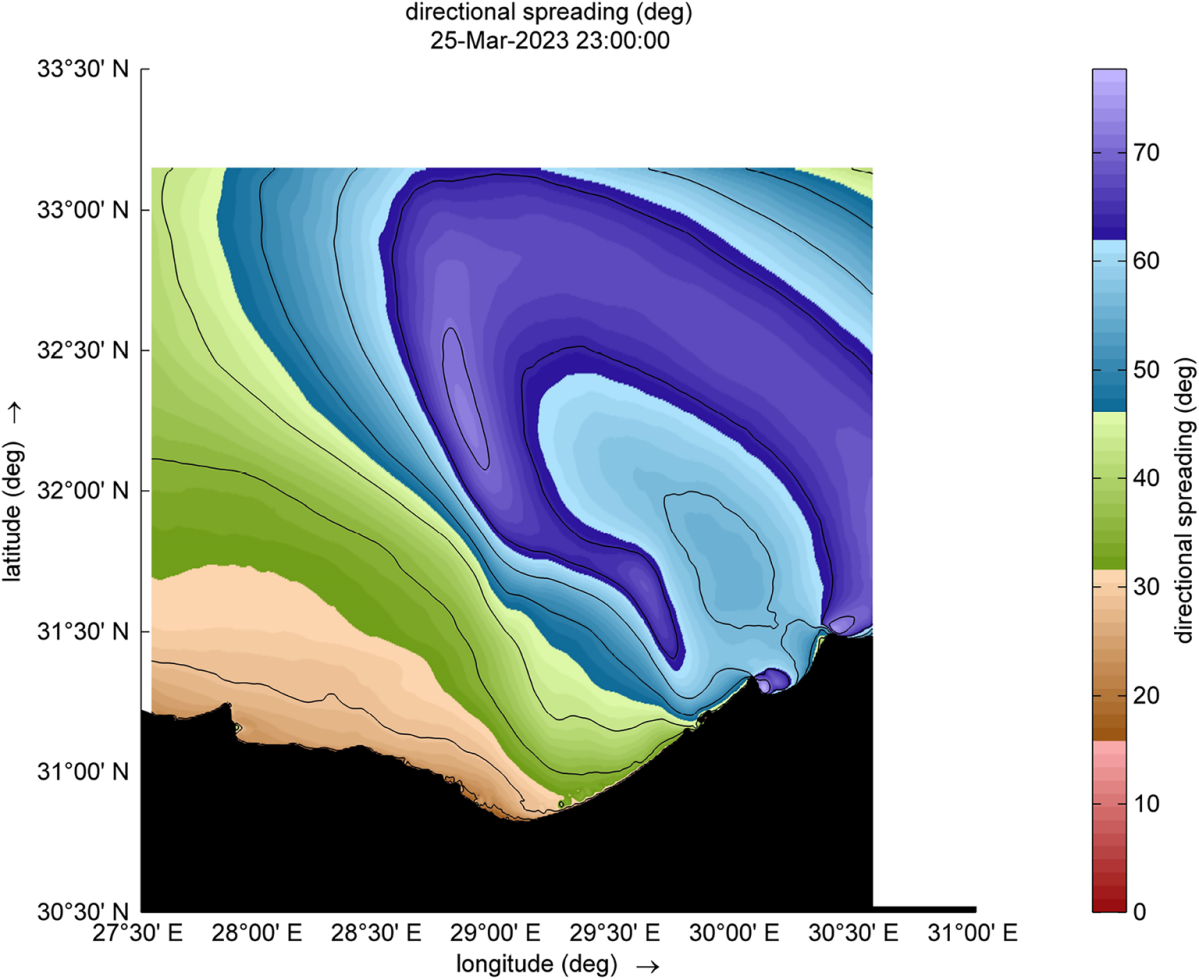
Directional spreading during the cyclone event.

The amount of energy transported from the wind to waves during this small-scale Mediterranean cyclone, especially during the two selected time steps (Figs. 9 and 10), reaches a maximum value of 12,080 W/m during [2023-03-25 17:00:00]. Such a huge amount has some explanation, the transfer of energy from wind to waves is achieved through two stresses. For this transfer to occur, these stresses must have harmonic components with the same wavelength as the waves. The first stress is exerted by the pressure acting on the surface water. The surface must move normal to itself, where the wind applies a horizontal force through differences in surface pressure. The second stress involves wind shear stress, which is exerted through the tangential orbital velocity on the potential flow (wave motion) and is associated with rotational flow. In the shallow water and surf-zone, an inconsistent wave field arises due to irregular wave set up. The dominant factor in this scenario is wave breaking, which governs the changes in energy transport and surface shear stress, driven by the intensity of the wave breaking. Furthermore, to preserve the transported energy, it is essential to minimize energy dissipation, as illustrated in Figs. 11 and 12, which are outcomes of the SWAN simulation. Furthermore, Figs. 13 and 14 show the results of directional spreading, reflecting the direction at which wave energy propagates. The average values of results range from 30° to 70°, indicating that a directional spreading of 70° means the wave energy is spread or distributed across a range of 70 degrees in terms of wave direction.

### Simulation of normal wave conditions (second scenario)

**Figure 15:**
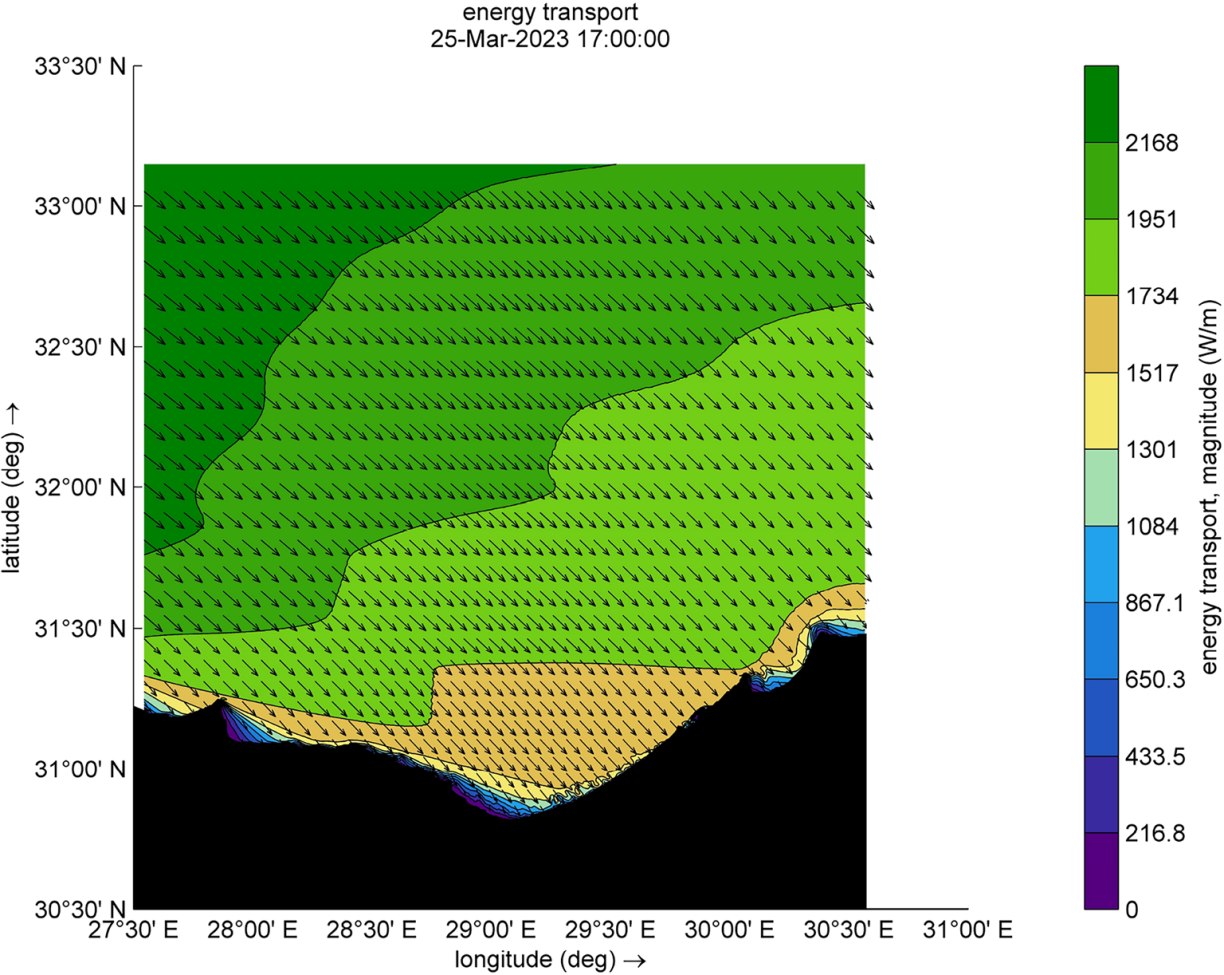
Distribution of energy magnitude and direction (indicated by black arrows).

**Figure 16:**
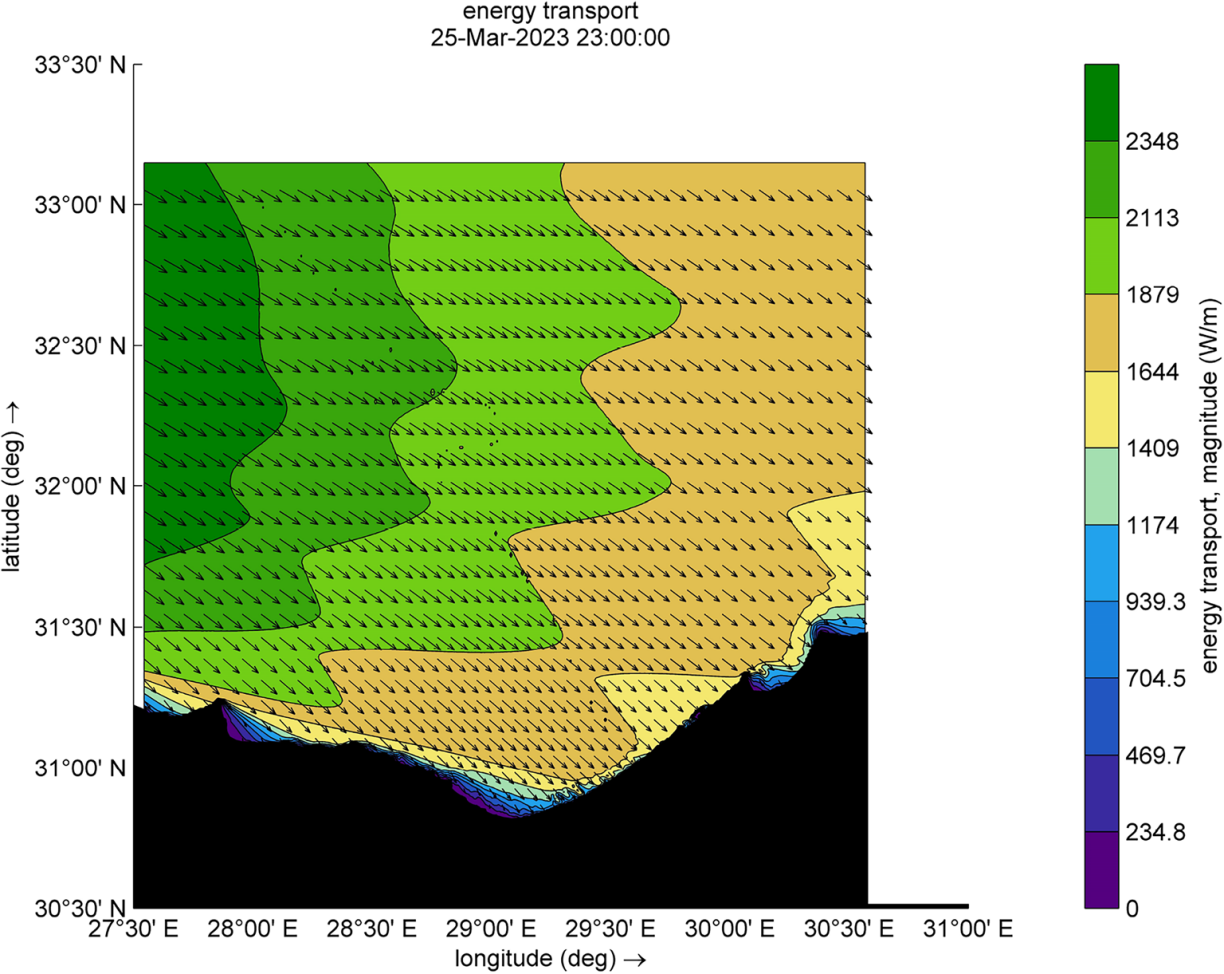
Distribution of energy magnitude and direction (indicated by black arrows).

This scenario illustrates the wave characteristics and energy transport along the study area. Additionally, two specific time steps [2023-03-25 17:00:00] and [2023-03-25 23:00:00] were selected, Figs. 15 and 16. The SWAN model is executed concurrently, characterizing the sea wave in this simulation by the significant wave height (Hsig) and wave direction. Wind forcing for this scenario was disabled, and the model run was executed without considering wind growth. The amount of energy transported, induced by forces other than wind, varies between 216 W/m and 2348 W/m.

### Calibration results

**Figure 17:**
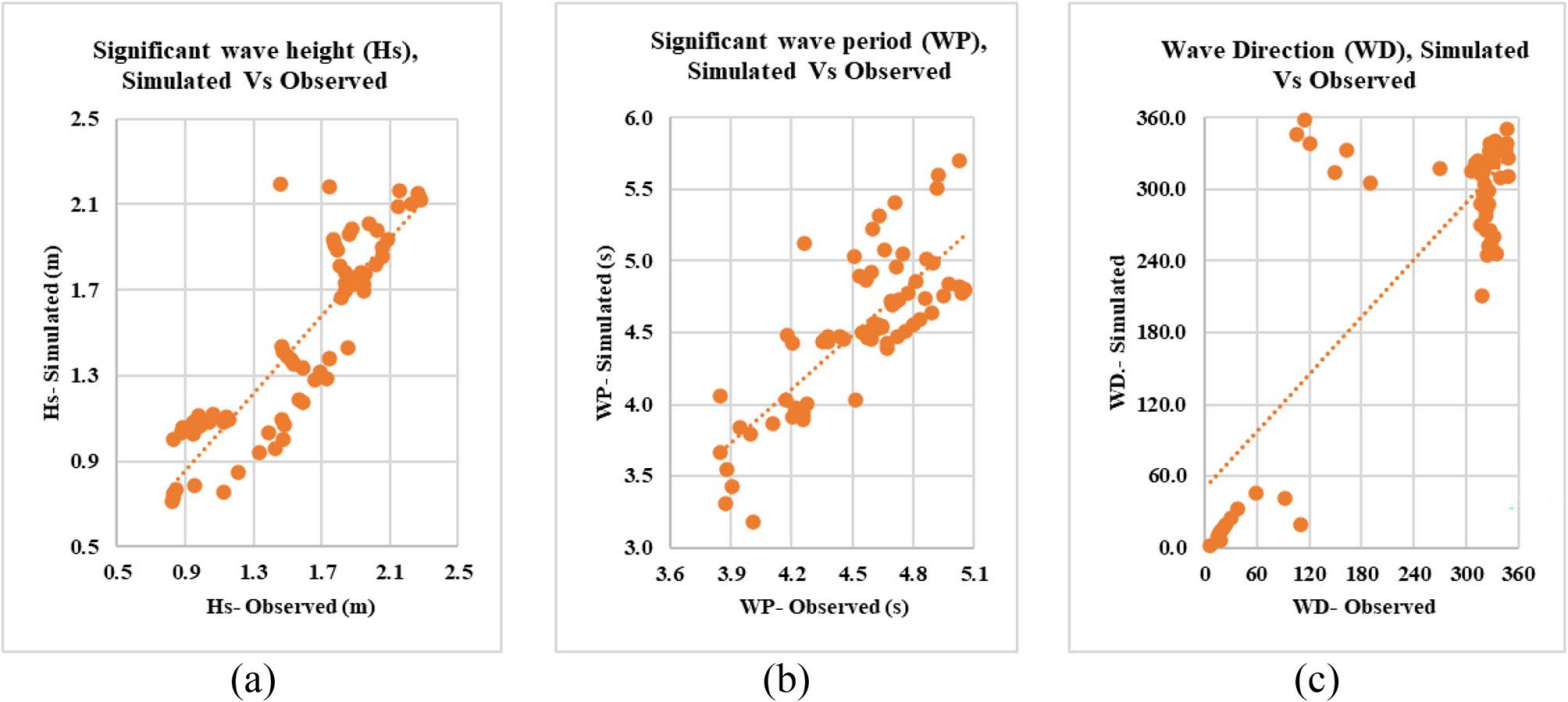
Scatter plots for the waves, depicting observations vs simulation results for the same geographical location, including significant wave height (**a**), significant wave period (**b**), and wave direction (**c**).

The analysis of wave observations from the fixed station at Abu Quir, as depicted in Table 2, provides valuable insights into wave dynamics during the cyclone event. The maximum significant wave height (Hs) reaching 2.28 m, alongside a corresponding wave period of 5.7 s, underscores the intensity of wave activity experienced. Despite the peak, the average Hs over the three-day cyclone duration remains slightly lower at 1.45 m. Comparison with SWAN model outputs, revealing an average Hs of 1.56 m and a wave period of 4.54 s, indicates a reasonably close match. Calibration results further support the model's accuracy, with a correlation coefficient of 0.8 for Hs, RMSE of 0.2 m, BIAS of 0.103m, MAE of 0.105, and Scatter Index (SI) of 0.167 suggesting minor discrepancies between observed and simulated data, Table 3. Furthermore, the scatter plot comparing simulation results to observations, as depicted in Fig. 17, demonstrates similarity. This analysis underscores the effectiveness of the SWAN model in capturing wave characteristics during extreme weather events, facilitating a deeper understanding of coastal dynamic.

**Table 2:** Descriptive statistics for the observations and the results of the model simulation (first scenario).

Descriptive statistics	Hs (m) Simulated	Hs (m) Observed	WP (s) Simulated	WP (s) Observed	WD (°) Simulated	WD (°) Observed
Mean	1.56	1.45	4.54	4.53	261.57	258.15
Standard error	0.05	0.05	0.04	0.06	13.73	13.33
Standard deviation	0.44	0.45	0.33	0.52	115.67	112.29
Minimum	0.83	0.71	3.85	3.18	6.39	1.39
Maximum	2.28	2.20	5.05	5.70	347.87	358.08

Where the significant wave height (Hs), wave period (WP), and wave direction (WD).

**Table 3:** Error statistic for the waves: observations and simulation results for the same geographical location.

Error statistic	Significant wave height (m)	Wave period (s)	Wave Direction
R	0.880	0.788	0.819
RMSE	0.238	0.325	67.696
BIAS	0.103	− 0.011	-3.368
MAE	0.105	0.011	3.368
SI%	0.167	0.073	0.266

### Validation results

**Figure 18:**
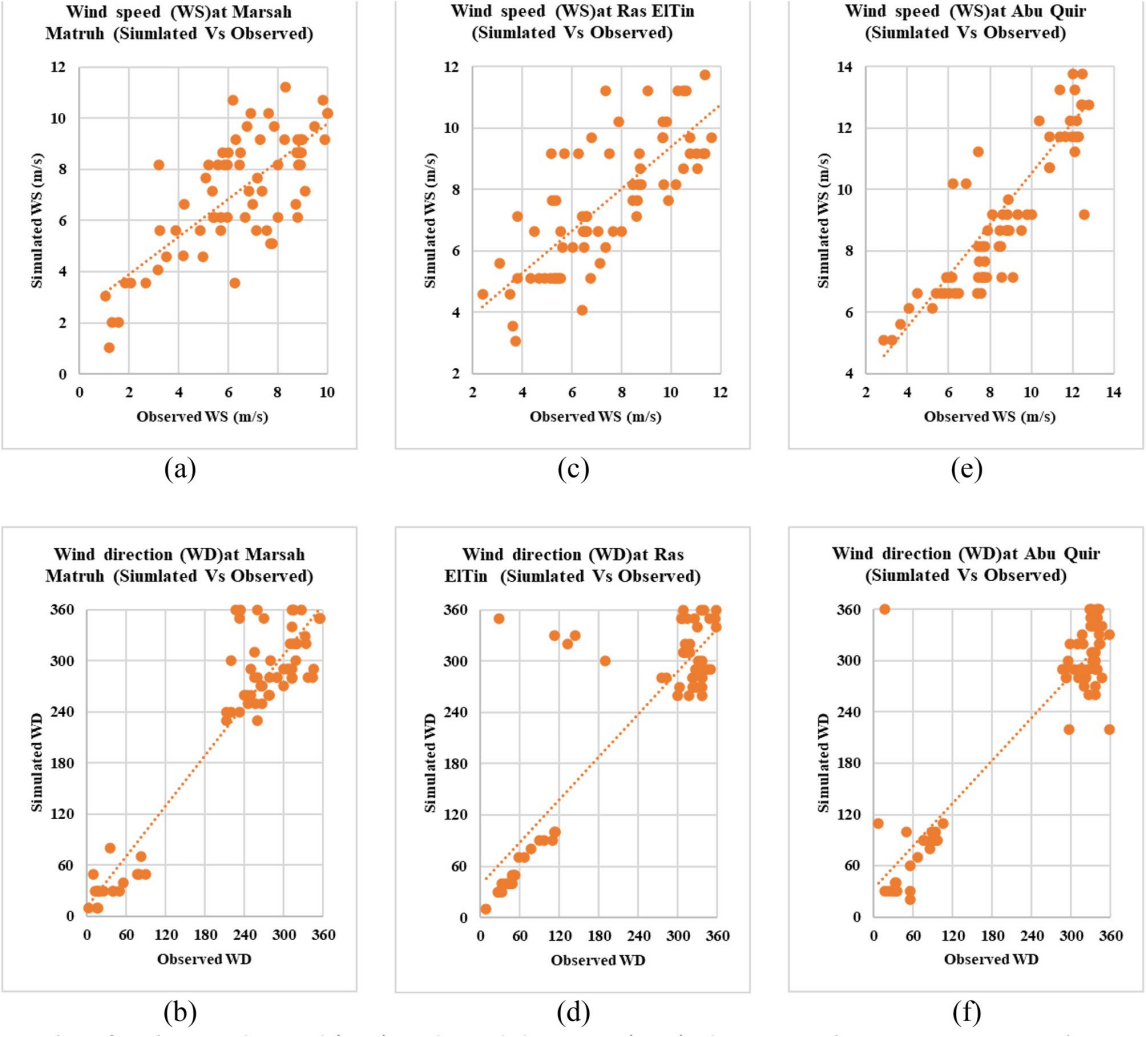
Scatter plots for the wind speed (WS) and wind direction (WD), depicting observations vs simulation results for three geographical locations; WS&WD at Marsah Matruh (**a**,**b**), WS&WD at Ras ElTin (**c**,**d**), WS&WD at Abu Quir (**e**,**f**).

The results of the quantitative analysis of wind observations derived from fixed stations located at Marsa Matruh, Ras El Tin, and Abu Quir, depicted in Table 4, show that the maximum wind velocity reached 11.22 m/s, 12.24 m/s, and 18.9 m/s, respectively. The average wind velocity at the three stations was 7.239 m/s, 7.69 m/s, and 9.48 m/s during the 3 days of the cyclone. Additionally, abnormalities in wind directions with variations in wind velocity from the same direction are noticed. Furthermore, For the same three geographic locations of the AWOSs and during the same period of investigation (from March 24, 2023, to March 26, 2023), six datasets of simulated wind data (wind velocity and direction) were exported from the SWAN model and used in the validation model. The quantitative analysis results are presented by some descriptive statistics and shown in Table 5. Results reveal that the maximum simulated wind speed occurred at Abu Quir, reaching up to 12.8 m/s, with an average velocity of 8.7 m/s. However, the minimum simulated speed was at Marsa Matruh, down to 1.07 m/s. The results of the error statistics for model validation are reported in Table 6. It is clear that the correlation coefficient (R), RMSE, MAE, and SI results at Abu Quir for wind speed are 0.761 m/s, 2.038 m/s, 0.752 m/s, and 0.215%, respectively. These results, along with scatter plots depicting similarity between observations and model simulation in Fig. 18, show a good fit between observations and model simulation.

**Table 4:** Descriptive statistics for the wind observations.

Descriptive statistics	Marsa Matruh		Ras ElTin		Abu Quir	
	Wind speed (m/s)	Wind direction (°)	Wind speed (m/s)	Wind direction (°)	Wind speed (m/s)	Wind direction (°)
Mean	7.239	229	7.692	219	9.48	231.527
Standard error	0.278	13.84	0.259	14.77	0.34	13.93
Standard deviation	2.36	117.4	2.205	125.4	2.86	118.2
Minimum	1.02	10	3.06	10	5.1	20
Maximum	11.22	360	12.24	360	18.9	360

**Table 5:** Descriptive statistics for the wind simulation.

Descriptive statistics	Marsa Matruh		Ras ElTin		Abu Quir	
	Wind speed (m/s)	Wind direction (°)	Wind speed (m/s)	Wind direction (°)	Wind speed (m/s)	Wind direction (°)
Mean	6.557	221	7.50	218	8.725	237.47
Standard error	0.287	13.29	0.291	15.53	0.309	15.229
Standard deviation	2.43	112.8	2.47	131	2.62	129.22
Minimum	1.07	3	2.39	9	2.856	7
Maximum	10.5	355	12.13	358	12.80	359

**Table 6:** Error statistic for the wind: observations and simulation results for the at three geographical locations.

Error statistic	Marsa Matruh		Ras ElTin		Abu Quir	
	Wind speed (m/s)	Wind direction (°)	Wind speed (m/s)	Wind direction (°)	Wind speed (m/s)	Wind direction (°)
R	0.757	0.944	0.770	0.873	0.761	0.910
RMSE	1.798	39.283	1.608	64.588	2.038	53.488
BIAS	− 0.682	− 8.222	− 0.188	− 1.343	− 0.752	5.952
MAE	0.682	8.222	0.188	1.343	0.752	5.952
SI %	0.248	0.171	0.209	0.294	0.215	0.231

## Conclusions and recommendations

In conclusion, this study unravels the intricate dynamics of Mediterranean cyclones, particularly focusing on the western Egyptian coast during a specific event which occurred in March 2023. The study area, employed the SWAN wave simulation model based on active wind growth field. The model was driven by spatially and temporally varying modified BOLAM data and underwent calibration and validation against real-time observations derived from already fixed stations. Given the abundance of studies focusing on sea surface hydrodynamics in this area, there is a need for a new approach utilizing SWAN. This approach emphasizes simulating energy transport, addressing issues related to damage followed by energy transfer. The investigation highlights the necessity for comprehensive research across both western and eastern Mediterranean regions. Cyclones in the Mediterranean significantly impact coastal hydrodynamics, influencing waves, and energy transfer. The utilization of the SWAN model in this study proves effective in simulating the complex interactions during the passage of a cyclone. Through two distinct scenarios, the research illustrates the profound effects of the cyclone on wave characteristics and propagation of energy transfer, with significant wave heights and directional spreading exhibiting notable variations. The wind field's asymmetry, coupled with changes in air temperature and atmospheric pressure, contributes to the cyclone's formation and intensity, offering valuable insights for understanding and predicting such events. Furthermore, while the model outcomes demonstrate strong comparability with findings from various researchers in the Mediterranean Sea, and the error statistics fall within acceptable ranges, a significant limitation lies in the scarcity of available calibration points. These points are predominantly located in nearshore and shallow areas, potentially amplifying errors.

As for recommendations, this study underscores the importance of continued research into Mediterranean cyclones, particularly in less-explored regions like the eastern Mediterranean and African coastlines. Enhanced monitoring and data collection in these areas can contribute to a more comprehensive understanding of cyclone behavior. Furthermore, coastal management strategies should incorporate the insights gained from this study, considering the potential impacts of cyclones on wave patterns and coastal erosion. Coastal communities, especially in vulnerable regions like Alexandria, should be equipped with early warning systems and adaptive measures to mitigate the risks associated with cyclones.

## Data Availability

Data are available upon request from the corresponding author.
